# Erythrocytosis and fatigue fractures associated with hepatoblastoma in a 3-year-old gelding

**DOI:** 10.4102/jsava.v90i0.1708

**Published:** 2019-03-28

**Authors:** Sharon Tirosh-Levy, Shmuel Perl, Beth A. Valentine, Gal Kelmer

**Affiliations:** 1 Department of Large Animal Surgery, Veterinary Teaching Hospital, Koret School of Veterinary Medicine, Robert H. Smith Faculty of Agriculture, Food and Environment, The Hebrew University of Jerusalem, Rehovot, Israel; 2 Department of Pathology, Kimron Veterinary Institute, Bet-Dagan, Israel; 3 Department of Anatomic Pathology, College of Veterinary Medicine, Oregon State University, Corvallis, United States

## Abstract

Hepatoblastoma was diagnosed in a 3-year-old Thoroughbred gelding presented with forelimb lameness with bilateral fatigue fractures of the proximal third metacarpal bones. An abdominal mass was detected on ultrasound examination of the abdomen. Absolute erythrocytosis was diagnosed after clinical and haematological evaluation. The fractured metacarpal bones were surgically removed but complications after surgery were fatal. The liver mass was diagnosed as a hepatoblastoma based on histology and immunochemical staining. The combination of hepatoblastoma and fatigue fractures has not been described previously in horses. A potential link between the hepatic and orthopaedic pathologies is hypothesised.

**Keywords:**

equine; erythrocytosis; hepatoblastoma; fatigue fracture.

## Introduction

Erythrocytosis is defined as a relative or absolute increase in the number of circulating red blood cells (RBCs) and is characterised by increased packed cell volume (PCV), haematocrit (HCT), RBC count and haemoglobin (HGB) concentration. Relative erythrocytosis is usually caused by dehydration or splenic contraction. Primary absolute erythrocytosis, or polycythaemia vera, is an idiopathic myeloproliferative disorder characterised by overproduction of all cell lines in the bone marrow and accompanied by normal to low concentrations of erythropoietin (EPO). Secondary absolute erythrocytosis is a result of EPO overproduction, which may be appropriate as compensation to systemic hypoxia, or inappropriate in cases of EPO secreting tumours or as part of a paraneoplastic syndrome (Lording 2008; Randolph, Peterson & Stokol 2010).

Stress or fatigue fractures are fairly common in young racehorses as a result of repeated high loading during training. Such injury occurs when the accumulation of micro-damages in the bone is faster than its repair or remodelling (Martig et al. 2014; Nunamaker, Butterweck & Provost 1990).

Equine primary liver tumours are rare, usually found in young animals, and classified as either hepatoblastoma or hepatocellular carcinoma based on the degree of cell differentiation. Only 12 cases of equine hepatoblastoma, of which only three cases were over 2 years of age, have been described in horses (Axon et al. 2008; Beeler-Marfisi et al. 2010; Loynachan et al. 2007; McFarlane, Sellon & Parker 1998). This case report describes the occurrence of erythrocytosis, hepatoblastoma and stress fractures in a 3-year-old Thoroughbred gelding and discusses a possible link between these conditions.

## Ethical considerations

The case describes treatment of a client-owned horse. All treatments were with the intention to help the horse, ameliorate its suffering and improve its medical status and its well-being. All treatments were given with the consent of the owner.

## Case presentation

A 3-year-old Thoroughbred gelding was presented to the Veterinary Teaching Hospital for surgical treatment of a stress fracture of the right proximal third metacarpal bone (MC3). The owners reported lameness as the only clinical abnormality and reported that prior to that, the horse was in training and raced successfully. Lameness and bright red mucous membranes were the only clinical abnormalities detected at the initial clinical examination.

**FIGURE 1:**
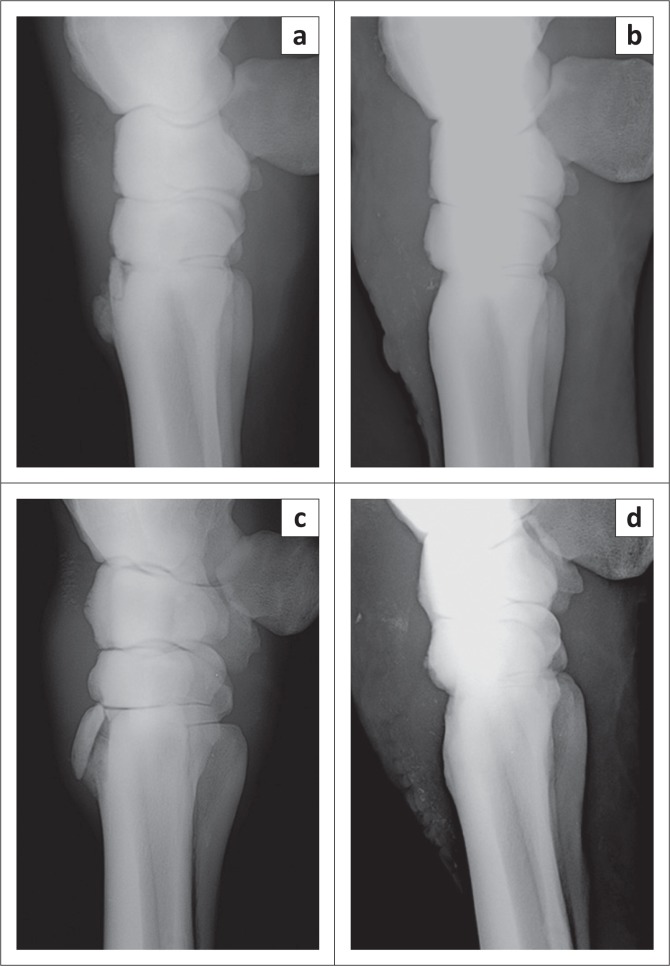
Computed radiographs of a 3-year-old Thoroughbred colt trained for racing. The left side depicts the left (a) and right (c) carpi, prior to surgery for removal of distinct, large, articular fatigue fractures at the proximal dorsal third metacarpal bone. The right side depicts the left (b) and the right (d) carpi after surgical removal of the fragments.

Radiographs of the forelimbs (FCR CAPSULA by Fujifilm, Tokyo, Japan) revealed bilateral fractures at the proximal dorsal aspect of both MC3. The fractures were almost symmetrical and involved the carpo-metacarpal joints (Figure 1).

The initial PCV was 62% (reference range [RR]: 30% – 45%) with total solids (TS) concentration of 6 g/dL (RR: 5–7.2 g/dL). The horse was not clinically dehydrated, and the creatinine concentration was lower than 0.5 mg/dL (RR: 0.9–2 mg/dL). After 24 L of intravenous fluid administration (via a 14G jugular catheter), the PCV was 60% with TS of 5.8 g/dL. The horse was discharged, and on return 1 week later, the PCV was 65%.

A complete blood cell count at this point revealed elevated RBC parameters (RBC: 14.12 × 10^6^/*µ*L, RR: 7.2–12 × 10^6^/*µ*L; HCT: 66.7%, RR: 31.1% – 50.5%; HGB: 24.9 g/dL, RR: 11.6–18. 9 g/dL), while white blood cell (WBC) and platelet counts were within normal limits. A serum biochemical profile disclosed increased serum activities of alanine amino transferase (ALT) (18.3 U/L, RR: 0–6 U/L) and gamma glutamyl transferase (GGT) (49.9 U/L, RR: 8–22 U/L), an elevated bilirubin concentration (8.4 mg/dL, RR: 0.5–2.3 mg/dL) and normal glucose concentrations (79/dL, RR: 50–107 mg/dL). Arterial blood gas parameters were within normal range except for a slightly low pressure of oxygen (PaO_2_: 83.4 mmHg). Indirect blood pressure obtained from the coccygeal artery was 107/84 mmHg.

Abdominal ultrasound (Mylab 30 model 7300 by Esaote, Genova, Italy, abdominal convex array transducer 25 MHz) suggested a large abdominal hyperechoic mass with ill-defined margins, just caudal to the diaphragm on the right side of the abdomen. Abdominocentesis yielded a transudate (TS: 1.4 g/dL) with few blood cells and no evidence of neoplastic cells. The mass was judged to be accessible for fine needle aspiration but was not sampled because the procedure lacked the owner’s consent.

A bone marrow aspirate from the sternum demonstrated a predominance of erythroid precursors (20:1 erythroid:myeloid ratio). A serum EPO concentration of 1.2 mIU/mL (no RR was established in the laboratory for equids, and the literature states normal ranges of 8–25 mIU/mL [McFarlane et al. 1998] or 0–9 mIU/mL [Jaussaud et al. 1994]). Reference samples analysed from two healthy mares with normal HCTs yielded an EPO concentration of 0.763 mIU/mL and < 0.6 mIU/mL.

At the request of the owner, bone fragments were removed from both MC3 under general anaesthesia. Surrounding proliferative bone was debrided. The horse was premedicated with xylazine hydrochloride (Tiazine, CEVA, Glenorie, NSW, Australia, 0.5 mg/kg bwt, IV) and butorphanol tartrate (Butomidor, Richter pharma, Wels, UK, 0.01 mg/kg bwt, IV). Anaesthesia induction was achieved with the administration of ketamine hydrochloride (Clorketam, Vetoquinol, Cedex, France, 2.2 mg/kg bwt, IV) and diazepam (Assival, Teva, Petach Tikva, Israel, 0.04 mg/kg bwt, IV) and maintained with isoflurane (Isoflurane, Teva, Petach Tikva, Israel) in 100% oxygen using positive pressure ventilation in a semi-closed breathing system (LAVC 2000 D, Eickemeyer, Germany). Intraoperative radiographs confirmed successful removal of bone fragments (Figure 1).

While recovering from general anaesthesia, the horse became hypoxaemic (PaO_2_ of 56.5 mmHg) and could not stand. Intravenous dextrose was administered (1 L of 10% dextrose), and the horse was placed in a sling, but even with sling support and head and tail ropes, the horse was still unable to stand. Six litres of blood was then removed through a catheter in the jugular vein. The horse rapidly improved, was able to stand and became more alert. After recovery, the PCV decreased to 53%.

Postoperative treatment consisted of bandaging, systemic antibiotics (Enrofloxacin, Vetmarket, Modi’in, Israel, 7.5 mg/kg SID PO) and anti-inflammatories (Phenylbutazone, Vetoquinol, Cedex, France, 4.4 mg/kg IV BID for 2 days followed by 2.2 mg/kg IV BID). The horse was discharged 5 days after surgery with a PCV of 57% and TS of 5.7 g/dL.

The use of fluoroquinolone (enrofloxacin) as a perioperative antibiotic in an elective surgery is contrary to recommended guidelines such as ‘Protect Me’ by BEVA (https://www.beva.org.uk/Home/Resources-For-Vets-Practices/Old-pages/Guidance/Protect-ME-Practice-Policy, last accessed September 2018). However, there was no available designated orthopaedic surgery suite, which compromised the level of cleanliness and sterility.

One month after discharge from the hospital, the horse returned weak and anorexic with cellulitis of the right forelimb. The animal collapsed and died soon after admission.

**FIGURE 2:**
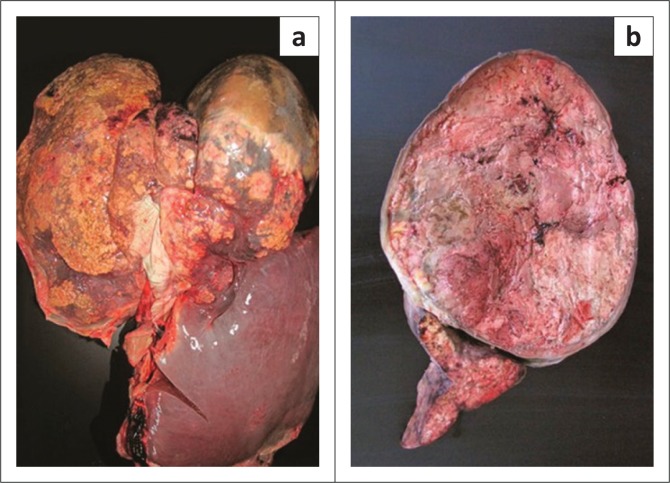
Post-mortem gross liver findings in a 3-year-old Thoroughbred colt that suffered from fatigue fractures (Figure 1) as well as consistent erythrocytosis. (a) Full view of the liver demonstrating a large round mass in the left medial lobe and multifocal masses in the left lateral lobe. The right liver lobe has normal appearance. (b) A transverse cut through the mass in the left medial liver lobe.

**FIGURE 3:**
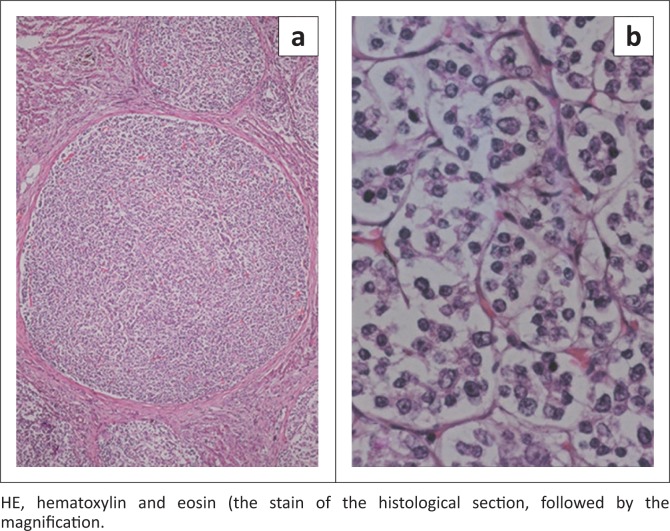
Histological sections of the liver from a 3-year-old Thoroughbred colt that suffered from fatigue fractures (depicted in Figure 1) as well as consistent erythrocytosis. (a) Multiple tumour nodules compressing adjacent hepatic parenchyma (*) (HE × 4). (b) Tumour cells are round, relatively homogeneous and form small nests supported by fine stroma (HE × 40).

Post-mortem examination revealed a large (approximately 20 cm) round firm mass in the left medial lobe of the liver. Numerous smaller masses of various sizes were present in the left lateral liver lobe (Figure 2). Both forelimb fracture sites appeared to have healed completely. Histological examination of the mass in the left lateral liver lobe showed marked disruption of the hepatic architecture by multiple variably sized masses, each composed of small relatively homogeneous cells with euchromatic nuclei, typically a single large central eosinophilic nucleolus, and a thin rim of finely granular to clear cytoplasm. Mitoses were rare. Tumour cells formed small nests surrounded by fine reticular-type stroma (Figure 3).

Immunostaining of tumour cells with CD117 (Dako, 1:500 dilution), S-100 (Dako, 1:400 dilution), CK-WSS (Dako, 1:500 dilution), CK AE1-AE3 (Dako, 1:200 dilution), Vimentin (Dako, 1:100 dilution) and Chromogranin (Dako, 1:1000 dilution) antibodies did not yield any positive reaction. Both cytokeratin (CK) ‘cocktails’ identified proliferative biliary-type epithelium in expanded portal areas of parenchyma adjacent to tumour nodules. Tumour stroma was positive for vimentin.

Based on clinical signs, gross pathology, histology and immunohistochemistry, the tumour was classified as an embryonic type of hepatoblastoma (Beeler-Marfisi et al. 2010).

## Discussion

Diagnosis of erythrocytosis relies on clinical findings and laboratory blood parameters. Clinical signs are usually attributed to blood hyperviscosity and to the underlying cause (Randolph et al. 2010). In this case, the horse was apparently healthy and the only initial clinical sign was red mucous membranes, accompanied by clinical pathology findings indicative of an increased RBC mass. The diagnosis of absolute erythrocytosis was made by ruling out dehydration and demonstrating the consistency of erythrocytosis over time.

Serum EPO concentration was not supportive of secondary erythrocytosis. Examination of bone marrow aspirates was not helpful in distinguishing between primary and secondary causes of erythrocytosis (McFarlane et al. 1998; Randolph et al. 2010). A cut-off value of 80 mmHg is usually used to diagnose hypoxaemia, which is usually accompanied by clinical signs of increased cardiac and respiratory rates or respiratory distress (Belli et al. 2011; Randolph et al. 2010). In this case, no clinical signs were observed but oxygen partial pressure was only slightly higher (83 mmHg) than the hypoxaemic cut-off. This may be attributed to blood hyperviscosity (Randolph et al. 2010). As this horse was a competing race horse before the initial diagnosis and did not demonstrate any clinical signs, cardiac or lung abnormalities were not likely, and the only probable differential diagnosis left was a neoplastic or paraneoplastic disorder. An abdominal soft tissue mass was indeed found ultrasonographically and liver-associated biochemistry parameters were elevated. However, as the owner was reluctant to perform further diagnostics, the definitive diagnosis of hepatic neoplasia was made only *post-mortem*. Erythrocytosis is the most common paraneoplastic phenomenon associated with hepatic neoplasia (Kew 2016) and has been reported in horses in at least three cases of hepatoblastoma and two cases of hepatocellular carcinoma (Beeler-Marfisi et al. 2010).

Erythropoiesis and bone homeostasis are finely coordinated regulated processes that occur in the bone marrow. When disease damages this balance, anaemia, polycythaemia or osteoporosis may occur (Eggold & Rankin 2018). It was shown in mice that polycythaemia causes bone loss and decreased osteoblast function both with and without excessive EPO production (Oikonomidou et al. 2016).

Hepatoblastoma is the most common liver tumour in children (Czauderna & Garnier 2018), but seldom reported in domestic animals. Veterinary reports of hepatoblastoma include a dog (Shiga et al. 1997), a cat (Ano et al. 2011), an alpaca (Watt, Cooley & Darien 2001), a bull, a sheep and 12 horses (Beeler-Marfisi et al. 2010). Most veterinary cases were diagnosed in neonatal or young animals [with the exception of an 8-year-old cat and a 13-year-old dog (Ano et al. 2011; Shiga et al. 1997)]. Half of the equine cases were reported in stillborn foals or neonates and only three cases were over 2 years of age. All of the horses showed various clinical signs (weight loss and inappetence) and the presenting complaint was usually non-specific. Some cases had other paraneoplastic signs including erythrocytosis, pleural effusion and ascites, as well as metastases in the lungs, bone, brain, meninges or abdominal cavity (Beeler-Marfisi et al. 2010; Cantile et al. 2001; de Vries et al. 2013; Lennox et al. 2000; Neu 1993; Prater, Patton & Held 1989). In this case, the horse reached 3 years of age with no apparent clinical signs and without interference with its athletic training prior to the reported lameness.

Based on tumour morphology and location, the primary differential diagnoses were hepatoblastoma, biliary carcinoma (cholangiocarcinoma) and hepatocellular carcinoma. Tumour morphology was also consistent with a metastatic gast-rointestinal stromal tumour, neuroblastoma and carcinoid. The final diagnosis of hepatoblastoma was made on the basis of a combination of morphological and histological features of the tumour.

Histological diagnosis of hepatoblastoma is challenging, with several subtypes, and definite diagnosis and classification can be established with the support of immuno-staining (Hiyama 2014; Schnater et al. 2003). The failure to detect CD117 ruled out a gastrointestinal stromal tumour and a negative S-100 reaction did not support neuroblastoma. Failure to detect cytokeratin within tumour cells ruled out biliary carcinoma, and the lack of chromogranin A ruled out a carcinoid tumour. Hepatoblastoma was the final diagnosis by ruling out all other relevant options. Hepatoblastoma cells will typically be alpha foetoprotein positive but, unfortunately, that antibody was not available for testing at the time.

The presenting complaint of the current case was of right forelimb lameness, and radiographs demonstrated bilateral proximal MC3 fragmentation involving the carpal joint. The symmetric nature of the fractures fits the assumption of fatigue injury. Material fatigue has undergone much study and refers to the process of degradation of material properties when a structure is repetitively loaded with forces that are less than the monotonic force required to cause catastrophic failure. Under continuous cyclic loading, microscopically small damage accumulates over time at areas of stress concentration and may eventually lead to catastrophic failure (Martig et al. 2014). Such failures are referred to as fatigue failures and in sport horses may eventually lead to catastrophic fractures. Fatigue fractures are directly related to bone mineral density (BMD) (Tóth et al. 2013). Fatigue fractures are common in racehorses, and most commonly they include MC3 stress fractures (‘bucked shins’), metacarpal or tarsal (MC3/MT3) condylar fractures, sagittal proximal phalanx fractures, proximal sesamoid fractures, carpal slab fractures, long bone (tibia, humerus and radius) fractures as well as fractures of the scapula and pelvis (Martig et al. 2014). Subchondral bone damage (mostly in the carpal and fetlock joints) is also characteristic in racehorses, which may lead to intra-articular fractures or focal joint surface injuries (Martig et al. 2014). Similar to this, in the current case, the fractures involved MC3 and were intra-articular, but involved the proximal MC3 and the carpo-metacarpal joint.

The effect of chronic liver disease on bone is well established in human patients. Bone mineral density decreases in liver patients, and they are prone to osteoporosis and pathologic fractures. This phenomenon was mainly described in cirrhotic patients and was typically named hepatic osteodystrophy (Guanabens & Pares 2010, 2011; Jadhav et al. 2000). Although the relationship between liver disease and decreased BMD leading to pathological fractures is well known, the aetiology and pathogenesis have not been fully elucidated. However, insulin like-growth-factor 1 (IGF-1) is manufactured in the liver and its decline is one of the first markers of liver dysfunction. Insulin like-growth-factor 1 is a major contributor to bone production, and thus, its loss in liver disease results in a decreased BMD (George et al. 2009; Guanabens & Pares 2010). A recent study reported that fractures occurred in 15% of children with hepatoblastoma. It is suspected to be a paraneoplastic syndrome, but no aetiology was suggested (Hartley et al. 1990; Towbin et al. 2018). In equines, there is a report of a yearling (https://jsava.co.za/index.php/jsava/editor/submissionReview/1708) with hepatoblastoma that suffered from bilateral bone cysts in the first phalanges (Axon et al. 2008). Unfortunately, in this case, as well as in the present one, no BMD analysis was performed to support this hypothesis.

## Conclusion

Cases of erythrocytosis are diagnostically challenging, especially in the absence of other clinical signs. Hepatoblastoma is a rare pathology and its description here, as an occult finding in a relatively mature and apparently healthy horse, is unique. The hypothesis of a potential link between the initial orthopaedic complaint and the final diagnosis warrants further investigation.
